# Trichodermin (4β-acet­oxy-12,13-epoxy­trichothec-9-ene)

**DOI:** 10.1107/S1600536808006168

**Published:** 2008-03-12

**Authors:** Shao-Yuan Chen, Chu-Long Zhang, Yu-Zhe Chen, Fu-Cheng Lin

**Affiliations:** a Insitute of Biotechnology, Zhejiang University, Hanzhou, People’s Republic of China; b College of Pharmaceutical Sicence, Zhejiang University, Hanzhou, People’s Republic of China

## Abstract

In the title natural product, C_17_H_24_O_4_, which is a very potent inhibitor of protein synthesis in mammalian cells, the five-membered ring displays an envelope conformation, whereas the two six-membered rings show different conformations, *viz*. chair and half-chair.

## Related literature

For related literature, see: Nielsen *et al.* (2005 ▶); Wei *et al.* (1974 ▶); Zhang *et al.* (2007 ▶). For details of ring puckering analysis, see: Cremer & Pople (1975 ▶).
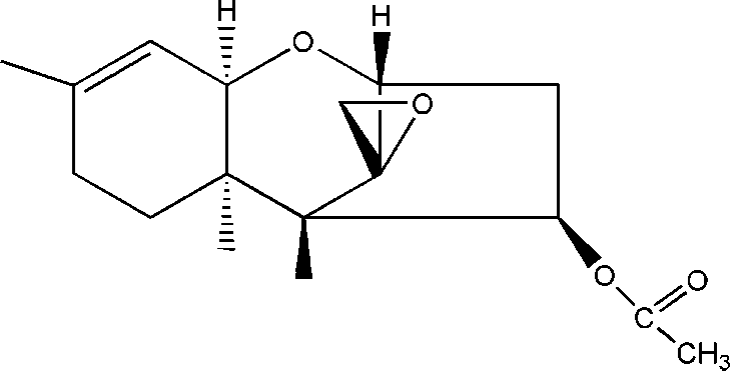


## Experimental

### 

#### Crystal data

C_17_H_24_O_4_
*M* *_r_* = 292.36
Orthorhombic, 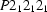

*a* = 7.0127 (3) Å
*b* = 8.4102 (3) Å
*c* = 26.2786 (10) Å
*V* = 1549.86 (10) Å^3^
*Z* = 4
Mo *K*α radiation
μ = 0.09 mm^−1^
*T* = 293 (2) K
0.41 × 0.40 × 0.37 mm

#### Data collection

Rigaku R-AXIS RAPID IP diffractometer
Absorption correction: none
14719 measured reflections
2046 independent reflections
1726 reflections with *I* > 2σ(*I*)
*R*_int_ = 0.027

#### Refinement

*R*[*F* ^2^ > 2σ(*F* ^2^)] = 0.036
*wR*(*F* ^2^) = 0.104
*S* = 1.11
2046 reflections
195 parameters
H-atom parameters constrained
Δρ_max_ = 0.18 e Å^−3^
Δρ_min_ = −0.13 e Å^−3^

### 

Data collection: *PROCESS-AUTO* (Rigaku, 1998 ▶); cell refinement: *PROCESS-AUTO*; data reduction: *CrystalStructure* (Rigaku/MSC, 2002 ▶); program(s) used to solve structure: *SIR92* (Altomare *et al.*, 1993 ▶); program(s) used to refine structure: *SHELXL97* (Sheldrick, 2008 ▶); molecular graphics: *ORTEP-3 for Windows* (Farrugia, 1997 ▶); software used to prepare material for publication: *WinGX* (Farrugia, 1999 ▶).

## Supplementary Material

Crystal structure: contains datablocks I, global. DOI: 10.1107/S1600536808006168/hb2704sup1.cif

Structure factors: contains datablocks I. DOI: 10.1107/S1600536808006168/hb2704Isup2.hkl

Additional supplementary materials:  crystallographic information; 3D view; checkCIF report

## Figures and Tables

**Table 1:** Hydrogen-bond geometry (Å, °)

*D*—H⋯*A*	*D*—H	H⋯*A*	*D*⋯*A*	*D*—H⋯*A*
C14—H14a⋯O2	0.96	2.58	2.936 (3)	102

**Fig. 1.**
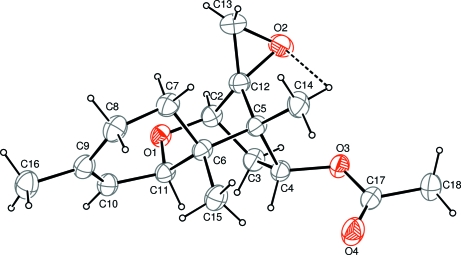
The molecular structure of (I) with 30% displacement ellipsoids (arbitrary spheres for H atoms), dashed line indicates hydrogen bonding.

## supplementary crystallographic information

### Comment

Trichodermin is a member of the 4β-aceoxy-12,13-epoxytrichothecene family (Nielsen *et al.*, 2005), which form a medically and economically important class of mycotoxins produced by fungi that spoil fruit and grain. Many studies (*e.g.* Wei *et al.*, 1974) show that trichodermin is a very potent inhibitor of protein synthesis in mammalian cells.

The molecular structure of (I) is shown in Fig. 1. The molecule contains two six membered rings, one five membered ring and one three membered ring. The five membered ring displays an envelope conformation with atom C12 at the flap position 0.705 (3) Å out of the mean plane formed by the other four atoms.

The O1-containing six-membered ring displays a chair conformation. The typical C9?C10 double bond length of 1.325 (3)Å suggests that C9 and C10 atoms are *sp*^2^ hybridized, which correlates with the larger C9—C10—C11 bond angle of 125.0 (2)° and C8—C9—C10 bond angle of 121.0 (2)° and a small C8—C9—C10—C11 torsion angle of 2.6 (3)°. A ring puckering analysis for the C9-containing six membered ring gave θ of 128.3 (2)° and φ of 206.9 (3)°, indicating a half-chair conformation (Cremer & Pople, 1975).

The C14-methyl group is attached to the bridgehead C5 atom and the small C14—C5—C12—O2 torsion angle of 38.4 (2)° allows a weak intramolecular C—H···O interaction (Table 1) to occur.

### Experimental

For morphological identification (Zhang *et al.*, 2007) cultures were grown on OA, PDA, and SNA media for 7–14 days at room temperature (293 K) under ambient daylight (Nielsen *et al.*, 2005). Microscopic observations and measurements were made from slides mounted in water. For metabolite production, the strains were inoculated onto PDA media and incubated for 10 days at 298 K in the dark. Selected strains were also cultivated in liquid media placed in a rotary shaker at 120 rpm for 10 days at 298 K in the dark. After cultivation, the bottles were stored at 253 K until extraction.

Liquid cultures were extracted with petroleum ether. The upper phase was filtered and evaporated *in vacuo*. Samples were then redissolved in petroleum ether to crystallize the crude product. Colourless chunks of (I) were recrystalized from n-hexane.

### Refinement

In the absence of significant anomalous scattering effects, Friedel pairs were merged; the absolute configuration was not determined.

The H atoms were geometrically placed (C—H = 0.93–0.98 Å) and refined as riding with *U*_iso_(H) = 1.2*U*_eq_(C) or 1.5*U*_eq_(methyl C). The methyl group was allowed to rotate, but not to tip, to best fit the electron density.

### Crystal data

**Table:**

C_17_H_24_O_4_	*D*_x_ = 1.253 Mg m^−^^3^
*M**_r_* = 292.36	Melting point = 319–320 K
Orthorhombic, *P*2_1_2_1_2_1_	Mo *K*α radiation λ = 0.71073 Å
Hall symbol: P 2ac 2ab	Cell parameters from 9452 reflections
*a* = 7.0127 (3) Å	θ = 3.2–27.0º
*b* = 8.4102 (3) Å	µ = 0.09 mm^−^^1^
*c* = 26.2786 (10) Å	*T* = 293 (2) K
*V* = 1549.86 (10) Å^3^	Chunk, colorless
*Z* = 4	0.41 × 0.40 × 0.37 mm
*F*_000_ = 632.00	

### Data collection

**Table:**

Rigaku R-AXIS RAPID IP diffractometer	2046 independent reflections
Radiation source: fine-focus sealed tube	1726 reflections with *I* > 2σ(*I*)
Monochromator: graphite	*R*_int_ = 0.027
Detector resolution: 10.0 pixels mm^-1^	θ_max_ = 27.4º
*T* = 291(2) K	θ_min_ = 3.0º
ω scans	*h* = −9→9
Absorption correction: none	*k* = −9→10
14719 measured reflections	*l* = −34→33

### Refinement

**Table:**

Refinement on *F*^2^	Hydrogen site location: inferred from neighbouring sites
Least-squares matrix: full	H-atom parameters constrained
*R*[*F*^2^ > 2σ(*F*^2^)] = 0.036	*w* = 1/[σ^2^(*F*_o_^2^) + (0.0625*P*)^2^ + 0.0793*P*] where *P* = (*F*_o_^2^ + 2*F*_c_^2^)/3
*wR*(*F*^2^) = 0.104	(Δ/σ)_max_ < 0.001
*S* = 1.11	Δρ_max_ = 0.18 e Å^−^^3^
2046 reflections	Δρ_min_ = −0.13 e Å^−^^3^
195 parameters	Extinction correction: SHELXL97 (Sheldrick, 2008), Fc^*^=kFc[1+0.001xFc^2^λ^3^/sin(2θ)]^-1/4^
Primary atom site location: structure-invariant direct methods	Extinction coefficient: 0.032 (3)
Secondary atom site location: difference Fourier map	

### Special details

**Table:**

Geometry. All e.s.d.'s (except the e.s.d. in the dihedral angle between two l.s. planes) are estimated using the full covariance matrix. The cell e.s.d.'s are taken into account individually in the estimation of e.s.d.'s in distances, angles and torsion angles; correlations between e.s.d.'s in cell parameters are only used when they are defined by crystal symmetry. An approximate (isotropic) treatment of cell e.s.d.'s is used for estimating e.s.d.'s involving l.s. planes.
Refinement. Refinement of *F*^2^ against ALL reflections. The weighted *R*-factor *wR* and goodness of fit *S* are based on *F*^2^, conventional *R*-factors *R* are based on *F*, with *F* set to zero for negative *F*^2^. The threshold expression of *F*^2^ > σ(*F*^2^) is used only for calculating *R*-factors(gt) *etc*. and is not relevant to the choice of reflections for refinement. *R*-factors based on *F*^2^ are statistically about twice as large as those based on *F*, and *R*- factors based on ALL data will be even larger.

### Fractional atomic coordinates and isotropic or equivalent isotropic displacement parameters (Å^2^)

**Table:**

	*x*	*y*	*z*	*U*_iso_*/*U*_eq_	
C14	0.6512 (4)	0.9319 (2)	0.36688 (10)	0.0589 (6)	
H14A	0.6793	1.0017	0.3390	0.088*	
H14B	0.7086	0.9727	0.3974	0.088*	
H14C	0.5156	0.9253	0.3714	0.088*	
C7	0.4588 (3)	0.6081 (3)	0.39880 (9)	0.0533 (5)	
H7A	0.3918	0.7071	0.4051	0.064*	
H7B	0.4180	0.5680	0.3660	0.064*	
C8	0.4061 (3)	0.4884 (3)	0.43987 (9)	0.0589 (6)	
H8A	0.4140	0.5397	0.4729	0.071*	
H8B	0.2752	0.4548	0.4348	0.071*	
C13	0.4905 (3)	0.7268 (3)	0.27735 (9)	0.0641 (6)	
H13A	0.4452	0.6423	0.2553	0.077*	
H13B	0.3912	0.7860	0.2947	0.077*	
O2	0.6596 (2)	0.81223 (19)	0.26194 (6)	0.0629 (4)	
C15	0.7438 (4)	0.6934 (3)	0.45039 (7)	0.0564 (5)	
H15A	0.6641	0.7780	0.4626	0.085*	
H15B	0.8732	0.7299	0.4482	0.085*	
H15C	0.7369	0.6051	0.4735	0.085*	
C5	0.7308 (3)	0.7668 (2)	0.35565 (7)	0.0433 (4)	
C18	1.1534 (4)	1.1714 (3)	0.34760 (9)	0.0627 (6)	
H18A	1.2106	1.2347	0.3739	0.094*	
H18B	1.0445	1.2262	0.3341	0.094*	
H18C	1.2444	1.1536	0.3209	0.094*	
C6	0.6749 (3)	0.6406 (2)	0.39726 (7)	0.0421 (4)	
C12	0.6697 (3)	0.7020 (2)	0.30416 (7)	0.0465 (5)	
C9	0.5325 (3)	0.3449 (2)	0.43982 (8)	0.0525 (5)	
C16	0.4718 (4)	0.2079 (3)	0.47267 (9)	0.0676 (6)	
H16A	0.5618	0.1226	0.4692	0.101*	
H16B	0.3478	0.1721	0.4622	0.101*	
H16C	0.4669	0.2415	0.5076	0.101*	
O4	1.0964 (3)	0.9811 (2)	0.41353 (6)	0.0741 (5)	
C17	1.0918 (3)	1.0153 (2)	0.36930 (9)	0.0506 (5)	
O3	1.0281 (2)	0.91593 (16)	0.33263 (5)	0.0502 (4)	
C4	0.9533 (3)	0.7633 (2)	0.34927 (8)	0.0440 (4)	
H4	1.0141	0.7309	0.3812	0.053*	
C11	0.7705 (3)	0.4800 (2)	0.38357 (7)	0.0419 (4)	
H11	0.9069	0.4892	0.3911	0.050*	
C10	0.6935 (3)	0.3431 (2)	0.41348 (8)	0.0486 (5)	
H10	0.7641	0.2495	0.4136	0.058*	
C2	0.8015 (3)	0.5625 (2)	0.29623 (7)	0.0461 (4)	
H2	0.7955	0.5252	0.2609	0.055*	
O1	0.7508 (2)	0.43802 (14)	0.33066 (5)	0.0462 (3)	
C3	0.9936 (3)	0.6396 (2)	0.30725 (8)	0.0497 (5)	
H3A	1.0438	0.6907	0.2770	0.060*	
H3B	1.0849	0.5610	0.3189	0.060*	

### Atomic displacement parameters (Å^2^)

**Table:**

	*U*^11^	*U*^22^	*U*^33^	*U*^12^	*U*^13^	*U*^23^
C14	0.0555 (13)	0.0392 (9)	0.0820 (14)	0.0064 (9)	0.0048 (11)	−0.0083 (10)
C7	0.0408 (11)	0.0516 (10)	0.0675 (13)	0.0032 (9)	0.0035 (10)	−0.0066 (10)
C8	0.0493 (12)	0.0608 (12)	0.0667 (13)	−0.0051 (11)	0.0101 (10)	−0.0109 (11)
C13	0.0542 (13)	0.0734 (14)	0.0648 (13)	0.0018 (12)	−0.0151 (11)	0.0026 (12)
O2	0.0649 (10)	0.0625 (9)	0.0612 (9)	0.0067 (8)	−0.0071 (8)	0.0092 (7)
C15	0.0640 (13)	0.0531 (10)	0.0520 (11)	−0.0085 (11)	0.0012 (10)	−0.0139 (9)
C5	0.0388 (9)	0.0374 (8)	0.0537 (10)	0.0026 (8)	−0.0021 (8)	−0.0079 (8)
C18	0.0740 (16)	0.0468 (11)	0.0672 (13)	−0.0104 (11)	0.0040 (12)	−0.0033 (10)
C6	0.0401 (10)	0.0383 (9)	0.0480 (10)	0.0012 (7)	−0.0008 (8)	−0.0095 (8)
C12	0.0442 (10)	0.0446 (10)	0.0508 (10)	0.0015 (8)	−0.0046 (9)	−0.0020 (8)
C9	0.0568 (12)	0.0509 (11)	0.0497 (10)	−0.0096 (10)	−0.0015 (10)	−0.0071 (9)
C16	0.0737 (16)	0.0677 (13)	0.0613 (13)	−0.0158 (13)	0.0082 (12)	0.0011 (11)
O4	0.1000 (14)	0.0670 (10)	0.0553 (9)	−0.0305 (10)	−0.0103 (9)	−0.0022 (8)
C17	0.0456 (11)	0.0478 (10)	0.0583 (12)	−0.0069 (9)	−0.0008 (9)	−0.0065 (9)
O3	0.0536 (8)	0.0426 (7)	0.0543 (8)	−0.0087 (6)	−0.0013 (6)	−0.0036 (6)
C4	0.0412 (10)	0.0386 (9)	0.0524 (10)	−0.0014 (8)	−0.0021 (8)	−0.0025 (8)
C11	0.0401 (10)	0.0399 (8)	0.0457 (10)	0.0007 (8)	−0.0017 (8)	−0.0067 (7)
C10	0.0531 (12)	0.0406 (9)	0.0521 (10)	−0.0014 (9)	−0.0032 (9)	−0.0061 (8)
C2	0.0498 (11)	0.0437 (9)	0.0447 (9)	0.0012 (9)	0.0008 (8)	−0.0087 (8)
O1	0.0520 (8)	0.0385 (6)	0.0481 (7)	−0.0008 (6)	−0.0003 (6)	−0.0100 (5)
C3	0.0452 (11)	0.0453 (10)	0.0586 (11)	0.0037 (8)	0.0051 (9)	−0.0071 (9)

### Geometric parameters (Å, °)

**Table:**

O1—C2	1.429 (2)	C15—H15B	0.9600
O1—C11	1.441 (2)	C15—H15C	0.9600
O2—C12	1.447 (2)	C5—C12	1.521 (3)
O2—C13	1.444 (3)	C5—C4	1.569 (3)
O3—C4	1.454 (2)	C5—C6	1.574 (3)
O3—C17	1.351 (2)	C18—C17	1.496 (3)
O4—C17	1.198 (3)	C18—H18A	0.9600
C9—C10	1.325 (3)	C18—H18B	0.9600
C14—C5	1.526 (2)	C18—H18C	0.9600
C14—H14A	0.9600	C6—C11	1.550 (2)
C14—H14B	0.9600	C12—C2	1.508 (3)
C14—H14C	0.9600	C9—C16	1.501 (3)
C7—C8	1.522 (3)	C16—H16A	0.9600
C7—C6	1.540 (3)	C16—H16B	0.9600
C7—H7A	0.9700	C16—H16C	0.9600
C7—H7B	0.9700	C4—C3	1.543 (3)
C8—C9	1.497 (3)	C4—H4	0.9800
C8—H8A	0.9700	C11—C10	1.495 (3)
C8—H8B	0.9700	C11—H11	0.9800
C13—C12	1.456 (3)	C10—H10	0.9300
C13—H13A	0.9700	C2—C3	1.523 (3)
C13—H13B	0.9700	C2—H2	0.9800
C15—C6	1.543 (3)	C3—H3A	0.9700
C15—H15A	0.9600	C3—H3B	0.9700
			
C5—C14—H14A	109.5	C11—C6—C5	108.57 (14)
C5—C14—H14B	109.5	O2—C12—C13	59.67 (13)
H14A—C14—H14B	109.5	O2—C12—C2	114.98 (16)
C5—C14—H14C	109.5	C13—C12—C2	125.02 (19)
H14A—C14—H14C	109.5	O2—C12—C5	117.80 (16)
H14B—C14—H14C	109.5	C13—C12—C5	128.51 (19)
C8—C7—C6	112.00 (18)	C2—C12—C5	103.23 (15)
C8—C7—H7A	109.2	C10—C9—C8	120.9 (2)
C6—C7—H7A	109.2	C10—C9—C16	122.2 (2)
C8—C7—H7B	109.2	C8—C9—C16	116.76 (19)
C6—C7—H7B	109.2	C9—C16—H16A	109.5
H7A—C7—H7B	107.9	C9—C16—H16B	109.5
C9—C8—C7	112.90 (18)	H16A—C16—H16B	109.5
C9—C8—H8A	109.0	C9—C16—H16C	109.5
C7—C8—H8A	109.0	H16A—C16—H16C	109.5
C9—C8—H8B	109.0	H16B—C16—H16C	109.5
C7—C8—H8B	109.0	O4—C17—O3	123.5 (2)
H8A—C8—H8B	107.8	O4—C17—C18	125.0 (2)
O2—C13—C12	59.88 (13)	O3—C17—C18	111.50 (19)
O2—C13—H13A	117.8	C17—O3—C4	116.79 (15)
C12—C13—H13A	117.8	O3—C4—C3	108.30 (15)
O2—C13—H13B	117.8	O3—C4—C5	111.98 (16)
C12—C13—H13B	117.8	C3—C4—C5	105.74 (16)
H13A—C13—H13B	114.9	O3—C4—H4	110.2
C13—O2—C12	60.45 (13)	C3—C4—H4	110.2
C6—C15—H15A	109.5	C5—C4—H4	110.2
C6—C15—H15B	109.5	O1—C11—C10	106.50 (15)
H15A—C15—H15B	109.5	O1—C11—C6	113.33 (15)
C6—C15—H15C	109.5	C10—C11—C6	113.11 (15)
H15A—C15—H15C	109.5	O1—C11—H11	107.9
H15B—C15—H15C	109.5	C10—C11—H11	107.9
C12—C5—C14	113.27 (18)	C6—C11—H11	107.9
C12—C5—C4	100.28 (16)	C9—C10—C11	125.0 (2)
C14—C5—C4	113.68 (16)	C9—C10—H10	117.5
C12—C5—C6	107.84 (15)	C11—C10—H10	117.5
C14—C5—C6	112.86 (16)	O1—C2—C12	109.28 (15)
C4—C5—C6	108.03 (16)	O1—C2—C3	114.30 (16)
C17—C18—H18A	109.5	C12—C2—C3	100.66 (14)
C17—C18—H18B	109.5	O1—C2—H2	110.7
H18A—C18—H18B	109.5	C12—C2—H2	110.7
C17—C18—H18C	109.5	C3—C2—H2	110.7
H18A—C18—H18C	109.5	C2—O1—C11	114.05 (13)
H18B—C18—H18C	109.5	C2—C3—C4	105.12 (15)
C7—C6—C15	109.60 (17)	C2—C3—H3A	110.7
C7—C6—C11	106.10 (16)	C4—C3—H3A	110.7
C15—C6—C11	108.99 (17)	C2—C3—H3B	110.7
C7—C6—C5	112.52 (16)	C4—C3—H3B	110.7
C15—C6—C5	110.89 (15)	H3A—C3—H3B	108.8

### Hydrogen-bond geometry (Å, °)

**Table:**

*D*—H···*A*	*D*—H	H···*A*	*D*···*A*	*D*—H···*A*
C14—H14a···O2	0.96	2.58	2.936 (3)	102
